# Identity crisis in pulmonary arterial hypertension

**DOI:** 10.1177/2045893217746054

**Published:** 2017-12-05

**Authors:** G. Ruffenach, S. Bonnet, S. Rousseaux, S. Khochbin, S. Provencher, F. Perros

**Affiliations:** 1 Groupe de recherche en hypertension pulmonaire et biologie vasculaire, 55973 Centre de Recherche de l’Institut Universitaire de Cardiologie et de Pneumologie de Québec, Laval University, QC, Canada; 2 55608 CNRS UMR5309, Inserm U1209, Université de Grenobles Alpes, Institut Albert Bonniot, Grenoble, France; 3 INSERM UMR_S 999, Université Paris–Sud, Laboratoire d’Excellence (LabEx) en Recherche sur le Médicament et l’Innovation Thérapeutique (LERMIT), Centre Chirurgical Marie Lannelongue, Le Plessis Robinson, France

**Keywords:** pulmonary hypertension, cancer hallmarks, tissues-specific gene, Darwinian evolution

## Abstract

Pulmonary arterial hypertension (PAH) shares many hallmarks with cancer. Cancer cells acquire their hallmarks by a pathological Darwinian evolution process built on the so-called cancer cell “identity crisis.” Here we demonstrate that PAH shares the most striking features of the cancer identity crisis: the ectopic expression of normally silent tissue-specific genes.

## Introduction

Pulmonary arterial hypertension (PAH) is a pulmonary vascular disease characterized by increased mean pulmonary arterial pressure (mPAP; >25 mmHg). This detrimental rise in pulmonary blood pressure is due to distal pulmonary artery occlusion driven by unregulated proliferation of pulmonary arterial smooth muscle cells (PASMC) and endothelial cells (PAEC) within the foci of complex vascular lesions. During the last decade, landmark studies demonstrated significant parallels between PASMC/EC in cancer and PAH,^1,2^ which elucidated key mechanisms of PAH pathogenesis.^1,3^ Studies describing the acquisition of cancer hallmarks by these vascular cells gave rise to the cancer paradigm of PAH.^2^

These hallmarks include an increased proliferation rate, evasion of growth suppressor, resistance to apoptosis, cellular energetic deregulation, angiogenesis in foci of complex vascular lesions, sustained inflammation, genetic mutations, and epigenetic modifications.^1,2^ Similar to what is observed in cancer, epigenetic modifications have been shown to predispose and participate in PAH pathogenesis.^4,5^ Indeed, germ line mutations in the bone morphogenetic protein receptor II (*BMPRII*)^4^ and potassium channel subfamily K member 3 (*KCNK3*)^6^ genes have been demonstrated to participate in PAH development. Furthermore, somatic mutations, single nucleotide polymorphisms, microsatellite instability, and aneuploidy have also recently been found to increase predisposition to PAH.^3,7,8^ For example, two sisters with heritable PAH carry the same missense mutation in Krüppel-like factor 2 (KLF2), a gene known to participate in nitric oxide vascular homeostasis.^9^ In another PAH patient carrying a BMPRII mutation, Aldred et al.^7^ identified a deletion in chromosome 13 resulting in the deletion of mothers against decapentaplegic homolog 8 (Smad-8). In both cases, these somatic mutations could participate in cancer and PAH pathogenesis. In addition, increased DNA damage followed by increased somatic mutation acquisition has recently been demonstrated in PAH.^10,11^ Finally, there is growing evidence for genome-wide epigenetic modifications in PAH that lead to chromatin remodeling and gene expression alterations. These modifications include varied expression of epigenetic modifiers and readers including histone deacetylases (HDACs)^12^ and BRD4,^13^ DNA methylation modifications,^14,15^ and microRNAs.^16^

**Fig. 1.**
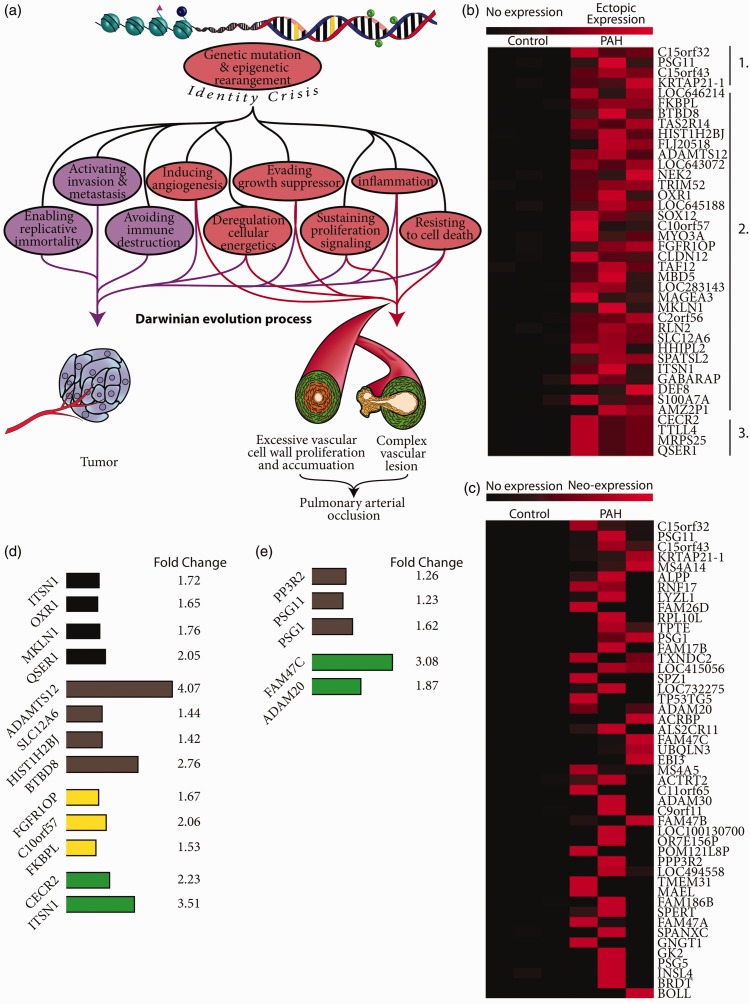
(a) Proposed model of identity crisis leading to PAH pathogenesis by a Darwinian evolution process. Genetic mutation and epigenetic rearrangement lead to aberrant gene expression and identity crisis development. In turn, identity crisis by a Darwinian evolution process gives rise to cancer cells hallmark acquisition and tumor development. In PAH, the same evolutionary process, with different selective pressure, would lead to PAH cancer hallmark acquisition, pulmonary arteries obstruction, and complex intimal lesions. (b) Ectopic expression of highly specific germline, placental, and embryonic genes in PAH lung. Ectopic expression heat map showing the ectopic expression in all human PAH lungs of highly specific germline, placental, and embryonic stem cell genes and their lack of expression in control lungs. Heat map has been divided into three groups: (1) placental and testis restricted expression; (2) germline cells predominant expression; (3) embryonic stem cells predominant expression (c) neo-expression of testis and placental restricted genes in PAH lung. Heat map illustrating the neo-expression in human PAH lungs of testis and placenta restricted genes and their absence of expression in control lungs. (d, e) Highly specific and restricted genes expression in previously performed microarray. (d) Aberrant expression of the same highly specific germline, placental and embryonic stem cell genes and (e) the neo-expression in human PAH lungs of testis and placenta restricted genes from microarray expression data of experiments previously performed in human PAH.^17^ These microarray expression analyses were performed on 12 lungs from end-stage PAH vs. 11 control patients (GSE 53408) (black); two primary isolated smooth muscle cells of PAH patients vs. two primary isolated control smooth muscle cells (GSE 21284) (gray); lungs from eight PAH patients associated with pulmonary fibrosis vs. 13 controls (GSE 15197) (yellow); lung from 18 PAH patients vs. 13 controls (GSE 15197) (green).

In cancer, genetic mutations and epigenetic modifications pave the way for the so-called cancer cell “identity crisis,” characterized by abnormal repression of cell-type-specific genes (from the differentiated phenotype) concomitant with the aberrant activation of “off-context” genes (normally expressed in other cell types or during different developmental stage) (Fig. 1a).^17^ These modifications in gene expression lead to a loss of the cells’ identity (i.e. phenotype) and the development of a full identity crisis (aberrant cell commitment). In cancer, the most striking example of this identity crisis is the activation of normally silent tissue-specific genes, such as male germline or placenta-specific genes.^17^ One hypothesis is that the development of this identity crisis provides a fertile ground for initiating a pathological Darwinian evolution process,^18^ leading to the acquisition of cancer hallmarks (Fig. 1a). In this view, after the first pro-cancerous event such as genetic and/or epigenetic modification, most altered cells would die from lethal genomic abnormalities. A few of them, however, would survive after accumulating specific “advantageous” genetic and epigenetic modifications leading to clonal expansion and growth. In cancer, this mechanism is supported by the monoclonal nature of cancer cells; whereas in idiopathic PAH (IPAH), clonal PAEC growth may take place within the plexiform lesions.^19^ Since PAH shares numerous hallmarks with cancer, we hypothesized that PAH could be characterized by a similar identity crisis and activation of normally silent tissue-specific genes. In order to investigate our hypothesis in the most elegant and irrefutable way, we aimed to identify aberrant activation of normally silent genes by employing a microarray analysis-based strategy that we recently developed.^17^

## Methods

### Patient population

In this study, we analyzed the lungs of one male and one female with IPAH and one female with PAH associated with systemic scleroderma (mean age = 47 years, mPAP =49.5 mmHg) and compared them to three non-PAH female lungs (mean age = 64 years). Individuals previously gave signed informed consent in accordance with Laval University and the IUCPQ Biosafety and Ethics Committee (CER20773).

### Microarray analysis

The detection of genes ectopically expressed from the transcriptomic analysis was carried out with a similar strategy as described by Rousseau et al.^17^ Briefly, using publicly available transcriptomic data from normal human tissue, a list of 506 genes with an expression restricted to the germline or the placenta tissue was defined. An extended list of genes was obtained by adding genes with a predominant expression in the germline, embryonic stem (ES) cells, or in the placenta on the basis of their mean expression level in one of these tissues being above a threshold defined as the mean expression in a series of 112 adult non-germline tissues + 3*standard deviations.^17^ The aberrant expressions of the abovementioned genes were systematically detected using the transcriptomic data of the samples from patients with PAH and a threshold value corresponding to the mean value in the three control samples + 3*standard deviations.

### Meta-analysis

In order to confirm the expression of genes found to be ectopically expressed in our microarray analysis, we investigated their expression by meta-analysis using the public Illumina microarray repository database, Nextbio.^20^ Nextbio allows user-friendly access to microarray data using a consistent analysis pipeline between datasets as described by Kupershmidt et al.^20^ We found three relevant microarray datasets in this microarray repository database; namely, GSE53408 comparing the lung gene expression of 11 controls to 12 patients with severe PAH, GSE21284 comparing primary cultured PASMC from two control to two PAH patients, and GSE15197 comparing the lung gene expression of 13 control to 18 PAH patients and eight patients with pulmonary hypertension associated with pulmonary fibrosis.

## Results

Our analysis revealed an aberrant expression of 40 genes in all three human PAH lungs (Fig. 1b). Among them, four genes (C15ofr32, PSG11, C15orf43, and KRTAP21-1) have normal expression strictly restricted to testis or placenta (Fig. 1b, Group 1), another four (CECR2, TTLL4, MRPS25, QSER1) have predominant expression in ES cells (Fig. 1b, Group 3), and the remaining 32 genes have predominant expression in germline cells (Fig. 1b, Group 2). Furthermore, considering only the genes whose expression is normally restricted to male germline or placenta, we observed the ectopic activation of 47 of these genes in at least one PAH patient (Fig. 1c).

These results were further confirmed by meta-analysis using Nextbio. Indeed, bio-informatics research in the Nextbio database revealed that, despite using a different microarray platform (Illumina vs. Affymetrix array), different approaches and different populations, three previous microarray studies also documented that 18 of the genes identified above have aberrant expression in PAH (Fig. 1d and 1e). Among these 18 genes, 13 of them were found to have altered expression in all three PAH patients (Fig. 1d) and five genes among the 40 genes with restricted tissues-specific expression (Fig. 1e). Presently, most of the genes we identified have no known function, highlighting the probable absence of a functional role in PAH pathogenesis.

## Discussion

To our knowledge, this is the first study demonstrating the aberrant activation of normally silent tissue-specific genes in the lungs of human PAH patients. Indeed, 23 of the altered genes have already been demonstrated in PAH, but this is the first time that a systematic investigation of off-context gene expression has been done in the context of PAH. This demonstration of an ectopic gene expression in PAH gives the first strong evidence of a cancer-like identity crisis in PAH. This also reinforces the potential role of epigenetic landscape modification in PAH pathogenesis^12–14,16^ as a mechanism to provide fertile ground for a pathological Darwinian evolution process.

Interestingly, while all the PAH patients we tested showed ectopic gene expression, they did not re-express the same set of genes. In our view, this differential re-expression is due to stochastic gene expression modification driven by the Darwinian evolution process. Thus, in this view, it is not the re-expression of a defined set of off-context genes which is of interest but rather the process of re-expression itself which reflects the underlying pathological mechanism that gives rise to the identity crisis. Nonetheless, a potential role for some of these ectopically expressed genes cannot be excluded, particularly for the genes we found expressed in all of our patients including embryonic stem cell genes which could participate in recruitment of progenitor cells known to play a role in PAH pathogenesis.^21^

Other studies are needed to investigate the specific role of these genes in a larger cohort of PAH patients. Indeed, the number of patients used in our study as well as their heterogeneity (e.g. sex, age, medication, and associated diseases) could lead to the generation of false-positive and false-negative results. Nonetheless, the generation of false-positive results is unlikely, considering the nature of genes investigated in the present study are epigenetically tightly locked in somatic cells.^17^ The generation of false-negative results cannot be controlled in our study setting; however, if this bias occurs, it will not nullify our finding of an identity crisis in PAH as false-negative results may only affect the number of genes found but not the demonstration of an off-context gene expression in PAH.

Taken together, our results demonstrate an off-context expression of normally silent genes which supports our hypothesis that an identity crisis is occurring in PAH. Much like in cancer, this off-context gene expression could pave the way for potential new predictive markers in PAH. In cancer, the ectopic expression of cancer testis antigens (CTAs), a class of immunogenic proteins with a testis- and tumor-restricted pattern of expression, are already use as predictive markers.^22^

In conclusion, our study brings the first evidence of an identity crisis occurring in the lungs of PAH patients by demonstrating the aberrant and off-context expression of normally silent, tissue-specific genes. In our view, this ectopic gene expression is due to a pathological Darwinian evolution process evolving on the fertile ground of genome-wide epigenetic modifications already suggested in PAH.^12–16^ This observation of an identity crisis in PAH strengthens the parallels that can be established between the PAH condition and cancer where a similar finding has been reported.
